# Intestinal and blood lymphograms as new diagnostic tests for celiac disease

**DOI:** 10.3389/fimmu.2022.1081955

**Published:** 2023-01-10

**Authors:** Garbiñe Roy, Fernando Fernández-Bañares, María Corzo, Sara Gómez-Aguililla, Carlota García-Hoz, Concepción Núñez

**Affiliations:** ^1^ Servicio de Inmunología, Hospital Universitario Ramón y Cajal, Instituto Ramón y Cajal de Investigación Sanitaria (IRYCIS), Madrid, Spain; ^2^ Department of Gastroenterology, Hospital Universitari Mutua Terrassa, Terrassa, Spain; ^3^ Instituto de Salud Carlos III, Centro de Investigación Biomédica en Red de Enfermedades Hepáticas y Digestivas (CIBERehd), Madrid, Spain; ^4^ Laboratorio de Investigación en Genética de enfermedades complejas, Instituto de Investigación Sanitaria del Hospital Clínico San Carlos (IdISSC), Hospital Clínico San Carlos, Madrid, Spain

**Keywords:** biopsy, blood, CD8 T cells, diagnosis, flow cytometry, lymphocytes, TCRγδ T cells

## Abstract

Accurate celiac disease (CD) diagnosis is still challenging for some specific patients or circumstances. Thus, much effort has been expended last decades focused on seronegative or low grade enteropathy CD and, especially, on enable early diagnosis of individuals on a gluten-free diet (GFD). We discuss here two diagnostic approaches based on immunophenotyping by flow cytometry that we expect to reduce the persistent low diagnostic rates and the common diagnostic delay. The intraepithelial lymphogram is based on determining the percentage of TCRγδ^+^ and surface CD3^-^ lymphocytes in the intestinal epithelium. The concomitant increase in TCRγδ^+^ and decrease in surface CD3^-^ intraepithelial lymphocytes has been termed the celiac lymphogram and has been proved to be discriminative in seronegative, low grade enteropathy and potential CD, as well as in most CD patients on a GFD. A blood lymphogram based on the analysis of activated gut-homing CD8^+^ T cells combined with a 3-day gluten challenge is also considered, which has shown high sensitivity and specificity to diagnose seropositive Marsh 1 and Marsh 3 CD in individuals following a GFD. In addition, flow cytometry can be extremely useful in cases of refractory CD type II to identify aberrant cells. Those approaches represent highly accurate methods for CD diagnosis, being simple, fast, highly reproducible and of easy implementation in clinical practice.

## 1 Introduction

Celiac disease (CD) is one of the best-known immune-related diseases, mainly due to the identification of gluten as the main environmental trigger and the understanding of some key features of the abnormal immunological response (1). Although the precise pathological processes governing the disease remain elusive, some of the major players are well identified (2). Thus, several populations of T cells show altered proportions in active disease and some of them even after gluten withdrawal. Immunophenotyping allows to select and count these immune-related cells according to their specific features offering an excellent opportunity for expanding diagnosis.

Flow cytometry is a very powerful tool for immunophenotyping. Since first developed in 1968 by Wolfgang Göhde from the University of Münster, flow cytometry has enormously expanded to provide extremely valuable information for many diseases and conditions and offer multiples possibilities of use in clinical practice. It is based on the measure of viable single cells in suspension that pass through a beam of light. These cells are combined with fluorochrome-labeled monoclonal antibodies that recognize and bind to specific antigen structures located, most frequently, on the cell surface, to identify the molecule of interest. Nowadays, the number of parameters that can be analyzed simultaneously has largely increased, allowing identifying specific cells depending on the expression of certain proteins (3). To facilitate data analysis, specific softwares have been developed, which provide different kind of graphics that accompany the result of each patient. Especially interesting are dot plots, which consider two parameters of study at the same time. This allows establishing a gating strategy, which constitute a basic principle in flow cytometry and it is determined by the sequential identification and refinement of the cell population of interest within a heterogeneous sample (4).

Nowadays, it is well established that gluten-specific CD4^+^ T cells recognize gluten peptide fragments presented by cognate HLA-DQ receptors, especially those previously deamidated by the enzyme transglutaminase 2 (TG2). Activation of these CD4^+^ T cells leads to an inflammatory process that results in the infiltration of the intestinal epithelium by diverse T cells, which constitute one of the hallmarks of CD, commonly as a first step towards the destruction of the epithelium. TCRγδ^+^ and TCRαβ^+^CD8^+^ (γδ^+^ and CD8^+^, respectively, hereinafter) represent the majority of the human intraepithelial T cell compartment, around 70%. The remaining 30% are linage negative innate lymphoid cells (ILCs) (hereinafter, surface CD3 negative, sCD3^-^). Both, T cells and sCD3^-^ cells, are present under homeostatic conditions with still poorly understood functions and altered proportions are observed in CD conferring an almost pathognomonic signature of CD condition. Notoriously, in most patients the high percentage of γδ^+^ intraepithelial lymphocytes (IELs) is also present years after gluten withdrawal. Moreover, gluten-specific CD4^+^ T cells in lamina propia and γδ^+^ and CD8^+^ IELs, all of them somehow involved in CD pathogenesis, have been described to be mobilized in CD patients on a gluten-free diet (GFD) after a short gluten challenge and allowed to be detected in blood three days after starting the challenge (5, 6). Based on the TCR sequences, γδ^+^ and CD8^+^ IELs were postulated to also play a role in the disease by developing antigen-specific responses, although contradictory results exist (5, 6). Importantly, we can use this knowledge to address the diagnosis of CD in subjects following a GFD, a still challenging issue.

CD can develop across the lifespan, but clinical and even analytical variables may be heterogeneous, leading to low diagnostic rates, especially in adulthood (7). Currently, a correct diagnosis requires solving a medical puzzle. The presence or absence of symptoms is nonspecific, and it is an unreliable indicator of CD (8). Response to a GFD is also nonspecific since there are patients with non-celiac gluten sensitivity (9). Celiac serology may be sufficient to make a diagnosis of CD when antibodies are present in high titer in both children and adults (8, 10), but low or borderline titers may be associated with no histological changes or minor changes of uncertain significance in duodenal biopsies (11). CD associated antibodies may be absent from the serum but subepithelial transglutaminase deposits may be detectable (12). Likewise, the interpretation of duodenal biopsies can be hindered by sampling error and cross-sectioning of tissue samples, and there is also a wide interobserver variability between pathologists (13). The characteristic histological features associated with CD are not pathognomonic and are shared by other conditions (14). Finally, assessment of celiac genetics (HLA-DQ2/8) is only useful to rule out the disease when it is non-compatible (11). Considering that adherence to a GFD constitutes an effective and safe treatment, accurate diagnosis leads to a great increase in the individual quality of life. However, the number of people following a GFD exceeds that of people with gluten-related disorders. Independently of the several underlying reasons, one relevant consequence is the impossibility of following a well-established clinical workflow for diagnosis. Mucosal healing, disappearance of circulating antibodies and resolution of clinical symptoms accompany the GFD and the routine diagnostic procedure include long gluten reintroductions that, besides commonly refused by patients, have not been standardized in clinical practice. Trials of novel celiac therapies also rely on a gluten challenge for the initial assessment of efficacy, but an accurate study design is not feasible without substantial data on the effects of a gluten challenge on currently available endpoints. A better understanding of the kinetics of serological and histologic changes that can occur during gluten challenge for CD is clearly needed, but CD patients are heterogeneous and linear correlations will not be probably found. Alternative possibilities or controlled gluten challenges following established protocols and providing objective measurements are still needed.

The following sections will discuss the clinical usefulness and accuracy of the immunophenotyping by flow cytometry for the diagnosis of CD using two already established procedures. Some methodological considerations of the presented tests will be also provided.

## 2 Intraepithelial lymphogram

**Figure 1:**
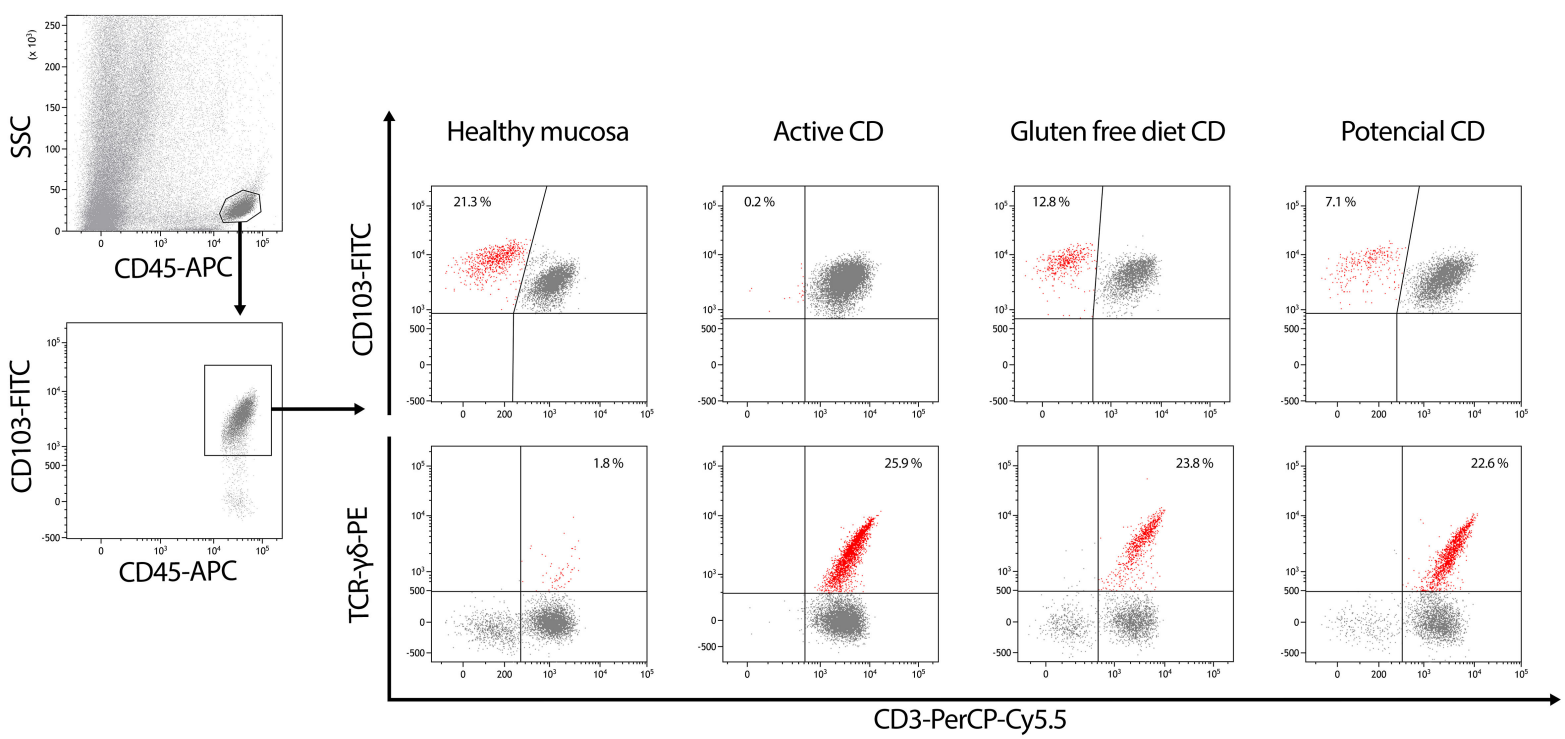
Gating strategy in the routine flow cytometric analysis of CD45^+^SSC^low^ cells isolated from the epithelium of duodenal biopsies. Surface CD3 (sCD3), TCRγδ and CD103 staining are selected in order to estimate the percentages of sCD3^-^CD103^+^ IELs (upper-left quadrants on top line dot plots) and TCRγδ^+^CD3^+^ IELs (upper-right quadrants on bottom dot plots). Intraepithelial lymphogram profiles represent an immunological signature of the different forms of celiac disease (CD).

An essential finding of celiac enteropathy is the increased number in total IELs in the duodenal mucosa (15), later characterized by an expansion of γδ^+^ and CD8^+^ IELs coupled to a decrease in sCD3^-^ IELs (16). The first description of an increased sCD3^+^CD4^−^CD8^−^ cell population in the intestinal mucosa of patients with active CD dates to 1983 (16, 17), which was later delineated as IELs bearing the γδ chains (18). Since then, several studies have confirmed the presence of high percentages of CD3^+^γδ^+^ IELs in the intestinal epithelium of CD patients, which seems to be permanent despite a GFD [reviewed in (19, 20)] and to have clinical relevance when compared with other types of enteropathy (*i.e.*, giardiasis, cow’s milk allergy), which show an increase in γδ^+^ IELs in a minority of patients with the condition that tends to be mild and transient (21). The same phenomenon was described in patients with potential CD (22, 23) (**Figure 1**). A more accurate quantification of the γδ^+^ subset became possible with the introduction of flow cytometry (24, 25). In 2002, a diagnostic algorithm for pediatric CD was proposed by Spanish researchers including the combined use of a high percentage γδ^+^ and a low percentage sCD3^-^ IELs, which had been termed the celiac lymphogram (19). However, its extended use has been limited to Spain, where it has been routinely used in clinical practice in the last decades and included in a national guide for early CD diagnosis in 2018 (26).

Nowadays, it is well known that the phenotyping of IELs by flow cytometry is of relevance in the diagnosis of CD. It constitutes a highly sensitive and specific complement to serology and histological examination for the diagnosis of CD, even in individuals with CD following a GFD who exhibit normal duodenal histology.

It is recognized that the celiac lymphogram does not provide a ‘gold standard’ for the diagnosis of CD, but the use of flow cytometry phenotyping of IELs can reinforce the diagnosis of CD when it is not clear-cut. The last European Society for the Study of Coeliac Disease (ESsCD) guidelines for CD stated, in the areas of uncertainty and future research, that further studies are needed to validate T cell flow cytometry and make it widely available for clinical use (8). Several studies related to this issue have been already published.

### 2.1 Children

Use of flow cytometric analysis of IELs as a routine diagnostic procedure in CD was introduced by Camarero et al. in the year 2000 in a pediatric series of 54 CD patients (24). This study evidenced the permanent imbalance in the density ratio of γδ^+^ *vs* sCD3^-^ IEL subsets that was almost pathognomonic of celiac conditions with independence of the clinical stage. It was termed celiac lymphogram. The authors designed a mathematical model to calculate the probability of suffering from CD that yielded a sensitivity of 94.4%, a specificity of 94.9%, a positive likely ratio (LR+) of 18.5 and a negative LR of 0.06. More recently, the same group has validated the diagnostic value of the lymphogram in an extensive pediatric series comprising 602 active CD patients, 92 on a GFD, 45 potential CD and 470 non-CD controls (27). When using stringent cut-offs of γδ^+^ (>15%) and sCD3^-^ (<6%), 90% of the active CD patients fitted in a complete “celiac lymphogram” with a 100% probability of disease. The remaining 10% of the active CD group displayed a “partial lymphogram” with lower disease probability. In this scenario, an isolated decrease on sCD3^-^ IELs yielded higher specificity and sensitivity for disease detection, with a disease probability of 76% *vs* 21% when the partial lymphogram was at expenses of an isolated increase of γδ^+^. When patients were on a GFD, the majority showed partial lymphograms at expenses of an isolated increase of γδ^+^>15% accompanied by a rising tendency on the density of sCD3^-^ IEL subset >6%, which lead to conclude that an increase on γδ^+^ IELs is the unequivocal marker of CD enteropathy while the size of sCD3^-^ IEL subset represents a useful marker to monitor diet compliance and mucosal integrity. The same was true for potential CD patients, where the increase of γδ^+^ was a constant hallmark at any time of the natural progression of this condition. γδ^+^ IEL counts has also been pointed out as a risk factor of overt disease in the largest cohort of potential CD patients so far published (28). By contrast, a persistent increase of sCD3^-^ IEL subset seemed to protect against progression to villous atrophy, being a good biomarker in the clinical management of potential CD.

Nowadays, lymphogram has become a routine diagnostic tool in many hospitals (29–38), useful not only in pediatric CD diagnosis but also in the much more complex scenario of adults’ gastroenteropathology, as discussed in the next paragraph.

### 2.2 Adults

There are several pivotal studies in adults addressing the role of phenotyping of IELs by flow cytometry in the diagnosis of CD. Most studies considered together children and adults, but some ones yielded results only for adults. In 2017, Valle et al. described a series of 161 CD patients, including 66 adults. Specifically in adults, sensitivity was 89% and specificity 96%, although similar results were obtained in children and adults (93% sensitivity and 96% specificity considering the whole data) (38). In 2019, Nijeboer et al. evaluated 95 adult patients with a mean age of 53 years, they only considered the increase in γδ^+^ cells (cut-off value >14%), showing a sensitivity of 66% and a specificity of 97% (37). It is noteworthy that the CD patients with normal γδ^+^ IELs in that study were significantly older than those with abnormal values. The authors argued that advanced age may be a factor preventing the characteristic increase of γδ^+^ IELs in CD for unknown reasons, and thus might explain the lower sensitivity found in that study. In this sense, the effect of sex, age and the degree of histological damage was evaluated (32) in a study including 169 CD patients with mean age 18.8 ± 1.5 years (range 1–83 years). Statistically significant differences in the median percentage γδ^+^ cells between age groups, including children, were not detected. Differences for sex, histological damage (Marsh 1 *vs.* Marsh 3) or titers of IgA anti-transglutaminase antibodies were also not observed either. Results showed 82% sensitivity, 100% specificity and 100% positive predictive value (PPV). However, considering that the number of CD patients and controls older than 61 was very small, the authors stated that further studies on this population group will be awaited. Another recent study evaluated 107 adult CD patients with a mean age of 60 years (35). Applying the described ‘IEL lymphogram’ criteria suggested by Ruiz-Ramírez et al. (≥8.5% TCRγδ^+^ and ≤10% sCD3^-^ IELs), 64% sensitivity and 92.5% specificity was observed. Rather than using single linear cut-offs for sCD3^-^ and γδ^+^ IELs, the authors identified a discriminant function as *%* *C* *D*3^+^ *I* *E* *L* *s*+2*x*(*%*γδ^+^ *I* *E* *L* *s*)>100. This function differentiated CD from control biopsies in the hypothesis generating group. The results were replicated in a validation group and found to be independent of histology in patients on long-term GFD up to 12 years (combined sensitivity, 98.5%; specificity, 98%). A recent study was focused on elderly patients (31). Eighty-seven patients ≥50 years were included at baseline (45 aged 50–59 years, 23 aged 60–69 years and 19 aged ≥70 years). A sensitivity of 95%, 89% and 87% was observed for each age group, respectively, using a cut-off value of γδ^+^>10%; and a sensitivity of 84%, 83% and 53%, respectively, for a cut-off value >14% (p=0.02; 50–69 *vs*. ≥70 years), with statistically significant difference between applying a cut-off of 10% or 14% (p=0.008). The median γδ^+^ count in the ≥70 years group was lower than in the other groups (p=0.014). Thus, maintaining a high specificity, results in adult populations suggest that sensitivity of the celiac lymphogram might decrease with increasing age above 70 years old.

As has been explained, the flow cytometry phenotyping of IELs shows a high specificity for CD diagnosis in adult patients and can be of diagnostic help in cases where diagnosis is not straightforward. A multicenter study was aimed on the diagnosis of seronegative villous atrophy (SNVA) (30). Sixty-seven patients with SNVA were included, 37 with CD and 30 with non-celiac villous atrophy. The celiac lymphogram was associated with a sensitivity of 87% and a specificity of 97% for CD. Among patients with a pre-test CD probability of 30%, post-test probabilities were 92% and 5% for positive and negative celiac lymphogram. The authors concluded that the celiac lymphogram was associated with a high level of diagnostic evidence either against or in favor of CD in patients with SNVA. The celiac lymphogram has also been shown to be a useful tool to consider Marsh 1 lesions as CD (29, 31, 32, 39–41). Using dermatitis herpetiformis as a model disease in which there are gluten-related symptoms despite of a non-atrophic enteropathy, even with negative celiac serology in a high percentage of patients, Popp and Mäki argue about the existence of a ‘celiac trait’, consisting in a Marsh 1 lesion, positive celiac genetics and increase in γδ^+^ cells, that should be identified and treated since they present clinical and histological remission after a GFD (42). Recently, a ‘low-grade celiac score’, including data on serology, intraepithelial lymphogram, HLA and histology, was derived statistically to identify patients likely to respond to a GFD and be diagnosed with Marsh 1 celiac enteropathy with a sensitivity of 86% and a specificity of 85%, and an AUC value of 0.91 (41). This score uses the γδ^+^ count, being the parameter that scores higher in seronegative patients. However, the diagnostic accuracy of the score was only maintained if there was a concomitant decrease of sCD3^-^ IELs, *i.e.*, when the celiac lymphogram was present (43). The ‘low-grade celiac score’ is thus a semiquantitative approach to characterize the ‘celiac trait’ described by Popp and Mäki, which may be useful in clinical practice.

Persistence of increased γδ^+^ cells after gluten withdrawal opens the possibility of diagnosing CD in patients who started on a GFD over several years and without any persistent histological changes on microscopic examination of duodenal biopsies, and without the need for undergoing a gluten challenge. Several studies focused on this aspect with promising results, but the studies were performed on small samples of patients, with the follow-time after a GFD not always described or only describing changes of mean values before *vs.* after the diet (24, 33, 35–37). Further studies on this item will be acknowledged.

### 2.3 Methodological aspects

IEL flow cytometry requires obtaining one single duodenal biopsy from the second portion of the duodenum, which is obtained in the same procedure as for histological analysis and commonly processed immediately, giving the results in the next four hours. However, the sample can be stored at 4 °C in complete medium 24 h before its processing and analysis (44).

This methodology is highly reproducible. In Spanish multicenter studies (30, 31) all participating centers used similar gating strategies to select both sCD3^−^ and TCRγδ^+^ cells, which were measured as CD45^+^CD3^−^CD103^+^ and CD45^+^CD103^+^TCRγδ^+^, respectively, over the total CD45^+^CD103^+^ cells. The authors performed comparative studies with samples in parallel between the hospitals and the concordance for both sCD3^−^ and TCRγδ^+^ cells was almost 100% in terms of absolute percentages [appendix A in (31)]. There were minor differences in the cut-offs for TCRγδ^+^ and sCD3^−^ cells used in the different participating centers, which imply slight variations in sensitivity and specificity between them, that is, the lower the cut-off, the higher the sensitivity and the lower the specificity, and vice versa. Some centers consider specificity and others sensitivity as priority. Thus, the quantitative individual values were interpreted in that studies with a single cut-off value that allowed for high specificity, thereby maintaining good sensitivity.

### 2.4 Refractory CD

Flow cytometry of intestinal IELs is the recommended method to identify the “aberrant” sCD3^-^ intracytoplasmic CD3 positive (icCD3^+^) IELs that expand in refractory CD type II (RCDII). Specifically, flow cytometry can precisely distinguish and enumerate the aberrant sCD3^-^ icCD3^+^ IEL subset and also the different CD8^-^ intraepithelial innate lymphoid cells and γδ^+^ cells, the latter being expanded in CD and RCD type I. A cut-off of 20% aberrant IELs has been proposed for a presumptive diagnosis of RCDII (45, 46).

More recent works include new flow cytometry markers in the assessment of this pre-malignant sCD3^-^ icCD3^+^ IEL subset. García-Hoz et al. showed that the increased intracytoplasmic fluorescent intensity (icCD3^+^) on the aberrant IEL subset predicts the risk of RCDII progression to enteropathy-associated T cell lymphoma (EATL), in good correlation with the presence of clonal TCR rearrangements (47). The authors conclude that the use of flow cytometry represents a powerful tool in the diagnosis and follow up of RCD: the enumeration of the sCD3^-^ icCD3^+^ aberrant IELs provides the initial presumptive diagnosis of RCDII, and the quantification of the icCD3 fluorescence intensity is of a prognostic value in determining the risk of clonality and progression to overt lymphoma. On the same line, Cheminant M et al. proposed NKp46, a Natural Killer receptor, as a useful biomarker for diagnostic and therapeutic stratification of gastrointestinal T lymphoproliferative disease (48).

A precise flow cytometry profiling of aberrant RCD IELs should help not only in the diagnosis but also in the clinical stratification and manage of patients, predicting survival and recommending therapies in this serious condition (49).

## 3 Blood lymphogram

### 3.1 Gut-homing T cells in blood

Detection of gluten-reactive T cells in peripheral blood remained elusive until CD patients on a GFD were challenged for three days aimed to identify CD relevant immunodominant gliadin epitopes (50). Maximum IFN-γ responses were observed on PBMCs collected six days after starting the 3-day gluten challenge by ELISPOT (Enzyme-linked immunospot) assay with overnight culture using a pool of antigenic peptides. This IFN-γ production mainly required CD4^+^ T cells and HLA-DQ, suggesting it was the result of HLA-DQ restricted CD4^+^ T cells specifically responding to gliadin. Particularly, the HLA-DQ2.5 immunodominant epitope was identified in α-gliadin 57-73, QLQPFPQPELPYPQPQS (p57–73 QE65). Abolishment of the IFN-γ response after depletion of the β7 integrin but not of αE (CD103), also suggested that those cells expressed the α4β7 integrin, which is associated with homing to the intestinal lamina propria (51). It was presumed that memory CD4^+^ T cells were causing the response. These cells most likely persist due to the presence of traces in the so-called gluten-free food or to the occasional gluten exposure that commonly occur in CD patients. It was assumed that they react to gluten presented by antigen presenting cells in mesenteric lymph nodes or gut associated lymphoid tissue, activate, clonally expand and *via* the thoracic duct reach peripheral blood, where they circulate until they migrate to the small intestine, which constitutes the effector site.

After identifying the gluten T cell epitopes and the transitory presence of gluten-specific T cells in blood, HLA-DQ2 tetramers could be generated to enable researchers to obtain directly the CD4^+^ T cells of interest (52, 53). Tetramers allow the study of antigen-specific T cell responses. They consist of multimerization using streptavidin of four biotinylated monomers of an antigenic peptide bound to MHC II molecules, which is necessary to obtain stable binding between them and the TCR. Streptavidin is coupled to a fluorophore for analysis by flow cytometry. Initially, DQ2-αI or DQ2-αII epitopes of α-gliadin were used as antigens. T cells detected with both DQ2-tetramer constructs were detected in all or the majority of patients. The presence of the gut-homing integrin β7 was confirmed by flow cytometry, but additionally, they were characterized as expressing CD28 and CD95. The state of differentiation was not homogeneous, but the markers observed suggested an effector memory phenotype (CD45RA^+^CD45RO^-^CCR7^-^CD27^-^) or a transitory state towards that phenotype (52).

In 2013, Han et al. demonstrated using time-of-flight mass cytometry (CyTOF) that CD8^+^ and γδ^+^ T cells could be also detected in blood after the 3-day gluten challenge in parallel to the described CD4^+^ T cell response (6). These cells expressed the integrin receptor CD103β7, which is indicative of migration to the gut intraepithelial compartment, and the activation marker CD38. Exhaustive analyses including a high number of markers suggested that these cells corresponded to effector memory T cells. Interestingly, it was observed that the detected cells resemble intestinal T lymphocytes present in mucosal biopsies of CD patients (5).

### 3.2 CD diagnostic tests

After the first works above described, IFN-γ ELISPOT and HLA-tetramers were proposed as possible tools for CD diagnosis (52, 54, 55). The last one was presented as faster and independent of the function of the cells, detecting naïve as well as memory T cells, and adding the possibility of a deeper characterization using flow cytometry. Interestingly, both approaches would enable diagnosis of individuals on a GFD. However, Brottveit et al. alerted in 2011 that tetramer test is quite laborious and the tetramer reagents have limited stability, making hardly probable the widespread use of the tetramer test (54). They reported that the use of tetramers was 100% specific and 85% sensitive for HLA-DQ2.5^+^ CD.

The findings of Han et al. (6) were used by our research group to try a new test to diagnose CD and thus we showed that the study of activated gut-homing CD8^+^ T cells by flow cytometry offered 95% specificity and 97% sensitivity for detecting seropositive CD (39). This test has a presumed easy implementation in clinical practice.

The three methodological approaches were based on a similar gluten challenge and the increase of IFN-γ, tetramers or activated gut-homing CD8^+^ T cells from day 0 to 6.

Although the three T cell subsets studied (gluten-specific CD4^+^, CD8^+^ and γδ^+^ T cells) show a similar kinetics after the 3-day gluten challenge, peaking at day 6-8 after the start of the challenge (6), the magnitude of the response observed was quite variable among patients. This has practical consequences when evaluating these cell responses by flow cytometry with diagnostic purposes. The number of γδ^+^ T cells in blood is low, making difficult to visualize the subgroup of γδ^+^ T cells mobilized after gluten challenge in subjects with a low response. Gluten-specific CD4^+^ T cells can be also hard to detect. CD4^+^ T cells recognize gluten peptides when presented by APC carrying the CD associated HLA-DQ molecules. The degree of immunogenicity depends on the specific gluten derived peptide/s and the individual HLA genetics. Some gluten epitopes elicit strong responses in most patients, which are called immunodominant epitopes. These are used to identify the CD4^+^ T cells of interest directly using HLA tetramers or indirectly by IFN-γ ELISPOT. However, the term gluten involves a heterogeneous group of proteins, a broad range of epitopes have been discovered and it is very likely that some epitopes are not represented in the antigen preparation used for research. The most extensively used peptide p57–73 QE65, is generally the immunodominant epitope in CD present in wheat, but this is not so in all patients. Accordingly, in the published works there are several cases of patients who respond to deamidated gliadin but they do not to the specific peptide used. The use of restricted sequences (antigen stimulation) may reduce the response to be observed although a cocktail of immunodominant peptides is used (56). Moreover, the use of antigenic peptides also needs to determine the optimal antigen concentration to obtain maximum responses.

The adequacy of flow cytometry to identify gluten-induced T cells was also evidenced by the work of Christophersen et al., who showed that gluten reactive CD4^+^ T cells could be identified from biopsy based solely on phenotypic markers (56).

There are some *a priori* limitations common to the three suggested approaches. First, all require at least a 4-week GFD period prior to the 3-day gluten challenge. It has not been determined the requirement of a very strict GFD, but nowadays, GFD can be easily monitored by determining gluten immunogenic peptides (GIPs) in stool or urine. GIPs result of the hydrolysis of gluten and have been shown to be sensitive and specific markers to detect ongoing gluten intake (57). Second, there is a high variability in the response among individuals; however, this is not a problem if extensive studies are performed in order to know the range of response and proper cut-offs for diagnosis are established.

### 3.3 CD diagnosis based on gut-homing CD8^+^ T cells

#### 3.3.1 Previous studies

In 2018, our research group showed that a 3-day gluten challenge induced the appearance of CD103^+^β7^hi^CD38^+^CD8^+^ T cells that could be detected in blood by flow cytometry with diagnostic purposes (58). The study included 15 CD patients and 35 non-CD controls following a GFD, all exposed to three days of gluten consisting in around 10-14 g consumed as 160-200 g of sliced white bread. Non-CD individuals were composed of 26 healthy volunteers, 3 patients with non-celiac gluten sensitivity and 6 disease controls. The described CD8^+^ T cell response was observed in all CD patients but only one healthy control. In 2021, we studied 22 CD patients and 48 non-CD controls (13 healthy subjects and 35 disease controls, 14 of this last group with a clinical response to a GFD) who underwent a similar gluten challenge protocol. The positive response was characterized by visualization of the studied T cell population after gluten challenge above a threshold (0.01% CD103^+^β7^hi^CD38^+^CD8^+^/total CD8^+^ T cells according to the studied patients) and a ratio day 6/day 0 ≥2. In this work, CD seropositive patients showing Marsh 3 or Marsh 1 at the diagnostic biopsy were included. The described test revealed 95% specificity and 97% sensitivity to identify seropositive CD. Only one patient displaying Marsh 1 and anti-endomysial antibodies but very low anti-TG2 antibody level (3.25 U/mL determined by ELISA with the commercial kit of Elia Celikey™, Phadia AB, Freiburg, Germany) when following a gluten containing diet, showed a negative T cell response. One patient with Marsh 3 did not show the response either, but he had been following a strict GFD for 25 years and the negative response to the gluten challenge was probably due to the high lapse of time, and thus, he was not considered for test accuracy calculations.

Interestingly, a positive T cell response was observed in the two non-HLA-DQ2.5 patients included (one CD patient showing DQ2.2 and one patient carrying HLA-DQ9). A patient carrying HLA-DQ8 had been previously reported to show the CD8^+^ T cell population after gluten challenge (6).

It has been described that the number of gluten-reactive T cells both in peripheral blood and in the small intestinal biopsy of CD patients positively correlated with the degree of histological intestinal damage (59). Accordingly, we observed that CD patients showing a Marsh 1 lesion at CD onset, displayed a T cell response to the short-term gluten challenge that can be distinguished from patients with atrophic lesions due to their lower magnitude. We did not observe correlation between the CD8^+^ T cell response and the duration of the GFD. However, this could be due to the differences among patients regarding the GFD compliance. With a good adherence, a decline in antigen-specific memory T cells as the time from the initial immunological response increases is expected. This is supported by the observed lack of the T cell response in the patient with a GFD for 25 years, which is concordant with previous observations (52, 60).

As previously described, the wave of T cells observed at day 6 after a 3-day gluten challenge appears independently of the clinical symptoms triggered by gluten.

Supporting our results, Leonard et al. observed an increased percentage of CD38^+^α4^+^β7^+^CCR7^-^CD45RA^-^CD8^+^ cells analyzing cryopreserved PBMCs by mass cytometry in CD patients on a GFD who received 10 g of gluten (55).

#### 3.3.2 CD8^+^ T cell-based diagnostic test: gluten challenge protocol and blood lymphogram

Most studies evaluating T cell responses to gluten challenge used gluten-containing sliced white bread (6, 50–52, 54, 61), although other gluten vehicles have been also successfully employed (55, 62, 63). We started using a bread-challenge, but replaced it by pure gluten, after ascertaining that the activated gut-homing CD8^+^ T cells were also detected (64), aimed to reduce ATIs and FODMAPs and the possible consequently associated clinical symptoms.

The amount of pure gluten was established in 10 g per day. Although some variations can be found in the literature, 10-20 g have been previously tested showing a positive T cell response. However, 3 g gluten per day was not enough to induce the response in some patients (55).

**Figure 2:**
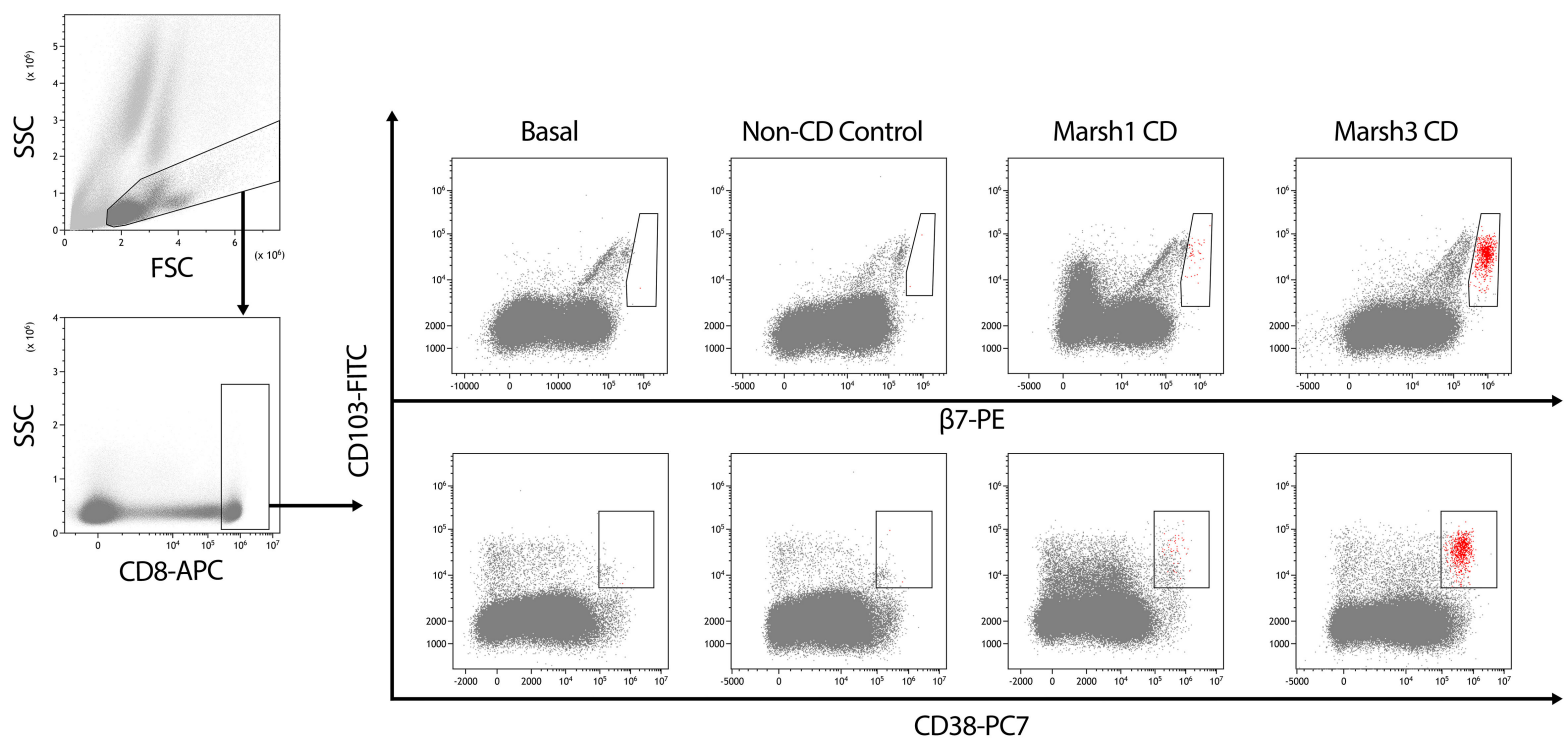
Gating strategy to analyze peripheral blood of individuals who underwent a 3-day gluten challenge. FSC vs. SSC gating was used to eliminate debris and select a gate containing the first population of study, which is later used to select CD8^+^ cells. Then, CD103/β7 (upper) and CD103/CD38 (bottom) dot plots are considered to select the cells being positive for the three respective markers, with the addition that only CD103^+^ cells with high expression of β7 are considered and shown in red in the CD103/CD38 plot. The blood lymphogram allows for the identification of different patterns in celiac disease (CD) and non-CD individuals. In CD, a higher response is observed in individuals with atrophy (Marsh 3) at onset. A total of 80,000 CD8 T cells is displayed in all the patients.

Considering the proved adequacy of a 3-day gluten challenge, the standardized protocol consists of 10 g of pure gluten that can be mixed with the liquid chosen by the patient (we recommend warm liquid yogurt to favor dissolution) and taken once a day (preferably early in the morning) for three consecutive days. This gluten challenge must be accompanied by two blood extractions: one before gluten intake and a second one 6 days later. Blood must be processed similarly both days. Briefly, whole blood is drawn and labelling with anti-human CD103, integrin β7, CD38 and CD8 monoclonal antibodies and erythrocyte lysis are performed. Data analysis consist in following a gating strategy to select the population of interest and obtaining the percentage of CD103^+^β7^hi^CD38^+^CD8^+^/total CD8^+^ T cells the two days of study. A different picture is obtained when analyzing CD or non-CD individuals (**Figure 2**). Non-CD subjects show a similar pattern the two days, most often characterized by the absence of the studied population. However, in some cases that population is observed but at a very low percentage or even at a considerable percentage but higher before gluten intake. The celiac blood lymphogram is characterized by a clear visualization of the CD103^+^β7^hi^CD38^+^CD8^+^ T cells at day 6 that must imply a higher percentage of those cells regarding total CD8^+^ T cells compared to the basal measure. Each laboratory must establish their own cut-off points, which could differ among centers depending on the preference on higher specificity or sensitivity, similarly to the exposed concerning the intraepithelial lymphogram.

#### 3.3.3 Advantages and limitations

One important advantage of the proposed methodology is that it uses whole blood, which can be employed directly for flow cytometry, requiring minimal sample manipulation. In addition, it allows sample processing and analysis one day after blood extraction showing high reproducibility, which enable transport between centers that can be done at room temperature using the same tube where blood was drawn. High reproducibility exists also when using different monoclonal antibodies. Finally, it must be underlined the speed to get the results: less than 3 hours from the second blood extraction (64, 65).

Importantly, a 3-day gluten challenge is well tolerated and it does not cause villous atrophy.

The main limitation when analyzing CD8^+^ T cells is that several gastrointestinal disorders can cause enteropathy characterized by increased CD8^+^CD103^+^ lymphocytes in the intestine (66). In fact, we also observed these cells in the blood of patients with gastroenteritis and with other conditions affecting the intestine such as small intestinal bacterial overgrowth. Therefore, it is crucial to compare the percentage of the studied lymphocytes before and after gluten intake. Other potential limitation, shared with the others methodological approaches based on the activation of memory T cells, is that a lack of response to the gluten challenge may exist in patients with more than 18 years in a GFD (39, 60). However, a second challenge at least 3 months later most probably gives a positive result in CD patients (60).

## 4 Conclusion

The diagnostic roadmap of CD most commonly needs to include different tools since none of the available methodological approaches discloses pathognomonic features. Taking an additional duodenal biopsy for flow cytometric analysis when samples for histological analysis are obtained allows determining the intraepithelial lymphogram and may provide useful information for decision-making. When interpreting the duodenal lymphogram, it is important to consider that:

- γδ^+^ IEL subset expands in all CD conditions, but not exclusively, and remains elevated long after gluten withdrawal. It is a biomarker of CD enteropathy.
- sCD3^-^ IEL subset is a dynamic lymphoid subset, highly represented in healthy mucosa and that almost disappears in active CD, but not exclusively, and tends to recover in healing mucosa. It is a marker of mucosal integrity.
- It is the concomitant evaluation of both IEL subsets, γδ^+^ and sCD3^-^, what defines the lymphogram.
- The more stringent the choice of cut-offs for IEL subset densities, the greater the discriminative power of the test. Although there is no consensus among the different groups on how to evaluate the duodenal lymphogram, there is common agreement on its usefulness in the diagnosis and follow-up of the diverse forms of CD.
- In case of suspicion of RCDII, the intracytoplasmic CD3 staining of the sCD3^-^ subtype is mandatory in order to identify and enumerate an aberrant sCD3^-^icCD3^+^ IEL subset.

Despite a GFD should not be encouraged for individuals with identified conditions different from CD or to get a healthier diet, it is a common practice. However, a firm diagnosis helps to avoid life-threatening complications. It must be considered that most of the changes associated with CD reverts after gluten withdrawal, making CD diagnosis on a GFD still challenging. Determination of the here described blood lymphogram represents a new diagnostic approach avoiding a long gluten reintroduction and the subsequent duodenal biopsy. Its correct interpretation needs to consider:

- Activated gut-homing CD8^+^ T cells can be present due to conditions others than CD. Therefore, increased percentage from day 0 to 6 must be observed to be indicative of CD.
- It must be accompanied of a 3-day gluten challenge, which has been established to provide a standardized protocol for gluten challenge to be used in clinical practice. Three days consuming 10 g of pure gluten daily represent the duration and amount of dietary gluten necessary to elicit a T cell measurable response.
- Patients to be studied can carry any HLA-DQ receptor. It is not HLA-DQ2.5-restricted.

Most laboratories in tertiary and even secondary hospitals are equipped with a flow cytometer for diagnostic purposes. Cellular suspensions can be easily obtained from the epithelium of the small bowel mucosa and blood. Thus, flow cytometry represents a simple, fast and cheap tool for diagnosis.

Both the IEL duodenal mucosa and the CD8^+^ T cell-based blood lymphograms provide reproducible results when samples are processed 24 h after being collected. This allows sample shipment to a different center for the analysis.

In summary, we present two methodological approaches that could be incorporated to clinical practice: the intraepithelial lymphogram always accompanying the histological analysis of duodenal biopsies and the CD8^+^ T cells determination in patients on a GFD who require a gluten challenge due to uncertain or lack of diagnosis and refuse a long-term gluten reintroduction.

## Author contributions

GR, FFB and CN draft the manuscript. MC, SGA and CG-H performed some experiments and designed the figures. All authors contributed to the article and approved the submitted version.
